# Therapeutic DBS for OCD Suppresses the Default Mode Network

**DOI:** 10.1002/hbm.70106

**Published:** 2024-12-25

**Authors:** Natalya Slepneva, Genevieve Basich‐Pease, Lee Reid, Adam C. Frank, Tenzin Norbu, Andrew D. Krystal, Leo P. Sugrue, Julian C. Motzkin, Paul S. Larson, Philip A. Starr, Melanie A. Morrison, A. Moses Lee

**Affiliations:** ^1^ Weill Institute for Neurosciences University of California San Francisco California USA; ^2^ Department of Psychiatry and Behavioral Sciences University of California San Francisco California USA; ^3^ Department of Radiology and Biomedical Imaging University of California San Francisco California USA; ^4^ Department of Psychiatry and Behavioral Sciences Keck School of Medicine of USC Los Angeles California USA; ^5^ Department of Neurology and Anesthesia and Perioperative Care University of California San Francisco California USA; ^6^ Department of Neurosurgery University of Arizona Tucson Arizona USA; ^7^ Department of Neurological Surgery University of California San Francisco California USA

**Keywords:** anterior limb of the internal capsule, deep brain stimulation, default mode network, DTI, fMRI, OCD, tractography

## Abstract

Deep brain stimulation (DBS) of the anterior limb of the internal capsule (ALIC) is a circuit‐based treatment for severe, refractory obsessive‐compulsive disorder (OCD). The therapeutic effects of DBS are hypothesized to be mediated by direct modulation of a distributed cortico‐striato‐thalmo‐cortical network underlying OCD symptoms. However, the exact underlying mechanism by which DBS exerts its therapeutic effects still remains unclear. In five participants receiving DBS for severe, refractory OCD (3 responders, 2 non‐responders), we conducted a DBS On/Off cycling paradigm during the acquisition of functional MRI (23 fMRI runs) to determine the network effects of stimulation across a variety of bipolar configurations. We also performed tractography using diffusion‐weighted imaging (DWI) to relate the functional impact of DBS to the underlying structural connectivity between active stimulation contacts and functional brain networks. We found that therapeutic DBS had a distributed effect, suppressing BOLD activity within regions such as the orbitofrontal cortex, dorsomedial prefrontal cortex, and subthalamic nuclei compared to non‐therapeutic configurations. Many of the regions suppressed by therapeutic DBS were components of the default mode network (DMN). Moreover, the estimated stimulation field from the therapeutic configurations exhibited significant structural connectivity to core nodes of the DMN. Based upon these findings, we hypothesize that the suppression of the DMN by ALIC DBS is mediated by interruption of communication through structural white matter connections surrounding the DBS active contacts.

## Graphical abstract

Therapeutic DBS suppressed BOLD activity within a distributed circuit implicated in OCD, and many of the suppressed regions are components of the default mode network (DMN). Moreover, there was a significant amount of structural connectivity between the estimated stimulation field of the therapeutic configurations and DMN.

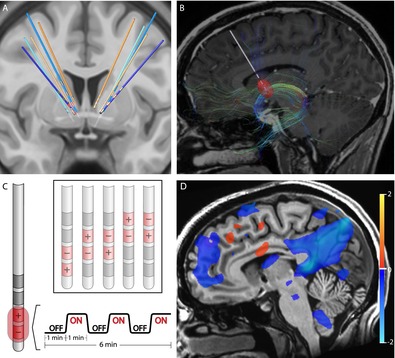

## 1 Introduction

Obsessive‐Compulsive Disorder (OCD) is a common psychiatric disorder characterized by intrusive anxiety‐provoking thoughts and repetitive behaviors. The symptoms of OCD are thought to result from aberrant activity within a cortico‐striato‐thalamo‐cortical (CSTC) network involving the orbitofrontal cortex (OFC), medial prefrontal cortex (PFC), and interconnected basal ganglia, and associated thalamo‐cortical circuits (Dougherty et al. 2018; Graybiel and Rauch 2000; Milad and Rauch 2012). Evidence‐based treatments for OCD include cognitive behavioral therapy and medications, such as serotonin reuptake inhibitors (Skapinakis et al. 2016). However, it has been estimated that approximately 10% of patients continue to have severe, debilitating symptoms that are not addressed by conventional therapies (Boschen and Drummond 2012).

Deep brain stimulation (DBS) is an invasive form of neuromodulation that has been used to treat severe cases of OCD (Goodman et al. 2020; Alonso et al. 2015; Denys et al. 2020). DBS involves direct electrical stimulation delivered through electrodes implanted in deep structures in the brain to modulate neural circuits. The most common DBS target for OCD is the anterior limb of the internal capsule (ALIC), which receives topographically organized connections from various components of the CSTC network (Baldermann et al. 2021; Haber, Yendiki, and Jbabdi 2021; Li et al. 2021; Hollunder et al. 2024).

However, the underlying mechanism by which DBS mediates its therapeutic effects remains unclear. This lack of mechanistic understanding is a barrier to addressing two important clinical limitations of the treatment: (1) Only 60% of patients respond to DBS at the ALIC target (Alonso et al. 2015; Denys et al. 2020) and (2) DBS programming to find the optimal configuration of stimulation contacts and parameters currently involves a complex, trial‐and‐error process guided by inconsistent subjective reports that can take months to years. For this reason, there is a need to identify biomarkers of target engagement that are tied to therapeutic efficacy. Such a biomarker could be used to predict treatment response and guide more efficient DBS programming.

Advances in DBS technology now allow for the safe acquisition of 3 Tesla (3 T) magnetic resonance imaging (MRI) data while DBS is cycled On and Off in bipolar configurations. With these advances, we can now map the network impact of DBS on whole brain activity using functional MRI (fMRI) blood‐oxygen‐level‐dependent (BOLD) imaging. The aim of this study was to determine the network effects of ALIC DBS by conducting fMRI while DBS was cycled On and Off in patients receiving DBS for severe, refractory OCD. We hypothesized that a common network related to OCD symptoms would be engaged specifically by therapeutic DBS configurations, and further that our estimated therapeutic DBS stimulation fields would exhibit strong structural connectivity with this network.

## 2 Methods and Materials

### 2.1 Subject Recruitment

Five patients treated with DBS for their severe, refractory OCD were identified from the University of California San Francisco (UCSF) OCD Clinic. In all patients, the DBS devices had been implanted under the FDA Humanitarian Device Exemption with bilateral leads targeting to the anterior limb of the internal capsule (ALIC) with the deepest leads typically targeted posterior to the anterior commissure within the bed nucleus of the stria terminalis (BNST). In all case, subjects had been receiving DBS treatment for at least 1 year. Only patients with a Medtronic Percept PC DBS stimulator (Medtronic, Minneapolis, MN), which is MRI conditional at 3 T, were screened for this study. Two participants were implanted with the older 3391 leads with wider spacing while the other three subjects were implanted with the more closely spaced 3387 leads. UCSF institutional review board and MRI safety committee approval was obtained prior to initiating recruitment. All subjects provided written informed consent for the study.

Three of the five subjects demonstrated marked clinical improvement of their OCD symptoms in response to DBS and were classified as treatment responders, defined as a greater than 35% reduction in their last Yale‐Brown Obsessive Compulsive Severity (YBOCS) compared to their pre‐surgical baseline. The remaining two subjects exhibited minimal clinical improvement for their OCD and related psychiatric symptoms and were classified as treatment non‐responders. In these subjects more than a year was spent trying to identify therapeutic settings without success, and their DBS devices are currently off (Table S1).

### 2.2 DBS Cycling Paradigm

We acquired 6‐min runs of fMRI data at 3 T using a block design where the DBS device was cycled On and Off for one‐minute intervals including 8 s to ramp stimulation up and down at the beginning of the DBS On and Off block, respectively. The Medtronic DBS Percept PC device is MR Conditional only in the bipolar configuration. During each fMRI run, stimulation was delivered in a bipolar configuration at a pair of adjacent electrode contacts on either the left or right brain lead. For each run, the active contact pair was chosen randomly from 12 possible configurations. Configurations were tested outside the scanner for tolerability before being trialed within the scanner. If configurations were not tolerable, usually because they elicited anxiety or somatic sensations, they were not tested in the scanner. Before and after each scan, the study clinician checked in on the participants to monitor whether participants were tolerating the cycling paradigm, and participants were notified that they could opt out of additional scans if they did not want to continue. Due to time constraints, only a subset of these configurations was tested in each subject. At the beginning of each scanning session, the DBS device was set in cycling mode, and the fMRI runs were timed to begin at the start of the Off cycle. Stimulation amplitude was set at the maximum cycling amplitude tolerated, which was either 5 or 6 mA for all patients.

Although the Medtronic Percept device is only labeled for MR imaging with bipolar stimulation, in treatment responders, the active treatment electrode configuration could be either a bipolar or monopolar configuration. For our fMRI experiments, if the responder's active configuration was a bipolar setting, this was considered the therapeutic configuration for that electrode. If the responder's active configuration was a monopolar setting, then the therapeutic configuration for that electrode was defined as the bipolar configuration for that electrode that shared the same anode as the active monopolar setting.

### 2.3 Imaging Acquisition

MRI scans (23 runs, 5 subjects, Table S2) were acquired on a 3 T whole body scanner in low specific absorption rate mode (Discovery MR750, GE Healthcare, Chicago, IL) with a 32‐channel receive head coil (Nova Medical). For all subjects, multiple runs of gradient‐echo fMRI data were acquired with the following parameters: TR/TE = 2 s/30 ms, voxel size = 3.75 × 3.75 × 4 mm, flip angle = 86°, FOV = 240 mm, in‐plane acceleration factor = 2, run length = 6 min. Manufacturer guidelines at the time allowed a maximum of 30 min of MRI scanning with the Medtronic Percept every 90 min. Thus, during each visit multiple 6‐min runs testing different configurations were acquired up to a total of 30 min of scan time.

Prior to entering the scanner, the participant's DBS device was programmed into a 1‐min On and Off cycling mode with an 8 s ramp. The scan was then manually initiated during the start of an Off period allowing for synchronization between the MRI and DBS systems. Between runs, the participant was removed from the scanner to an MRI safe zone while lying on the detached scanner bed with their head in the same position so the study clinician could change the DBS settings before being returned to the MRI. Some participants returned for multiple visits over separate days to acquire additional scans with different configurations.

For three subjects (one responder, two non‐responders), we collected pre‐surgical diffusion weighted images (DWI) (3 T, 55 direction, *b* = 2000 s/mm^2^). For two of the subjects (both responders), no pre‐surgical DWI was available, so we collected post‐implant T1‐weighted (T1w) structural MRI and DWI (3 T, 29‐direction, *b* = 1000 s/mm^2^) for these participants. We have previously demonstrated that tractography using post‐implant DWI is feasible and reproduces results from pre‐implant DWI (Basich‐Pease et al. 2023).

### 2.4 Image Preprocessing and Denoising

T1 and fMRI data preprocessing was performed with fMRIPrep 21.0.1 (Esteban et al. 2019), a standard preprocessing pipeline based on Nipype 1.6.1 (Gorgolewski et al. 2011), which uses Nilearn 0.8.1 (Abraham et al. 2014) for many internal operations. Anatomical preprocessing generated a subject‐specific T1w reference template for registration of fMRI images to T1w (subject) and MNI spaces. In brief, the anatomical preprocessing steps included the following: T1w images from all scanning sessions for an individual subject were bias field corrected with N4BiasFieldCorrection (Abraham et al. 2014) in ANTs 2.3.3 (Avants et al. 2008) and averaged to generate a subject‐specific anatomical reference image using mri_robust_template (FreeSurfer 6.0.1) (Reuter, Rosas, and Fischl 2010). The T1w reference was skull‐stripped using FAST (FSL 6.0.5.1) (Zhang, Brady, and Smith 2001) then normalized to the MNI152NLin6Asym standard space via nonlinear registration with ANTs.

For each fMRI run, skull‐stripped and non‐skull‐stripped BOLD reference volumes were co‐registered to subject (T1w). Head‐motion parameters with respect to the fMRI reference (transformation matrices and six corresponding rotation and translation parameters) were estimated before any spatiotemporal filtering using mcflirt (FSL 6.0.5.1) (Jenkinson et al. 2002). BOLD runs were slice‐time corrected to 0.98 s (mean of slice acquisition range 0–1.96 s) using 3dTshift from AFNI (Cox 1996; Cox and Hyde 1997). The slice‐time‐corrected BOLD time‐series were resampled onto their original, native space by applying the transforms to correct for head‐motion. The BOLD reference was then co‐registered with six degrees of freedom to the T1w reference using bbregister (FreeSurfer) which implements boundary‐based registration (Greve and Fischl 2009). Framewise displacement (FD) was calculated based on the preprocessed BOLD using two formulations, absolute sum of relative motions (Power et al. 2014), and relative root mean square displacement between affines (Satterthwaite et al. 2013), using Nipype (Gorgolewski et al. 2011). Frames that exceeded a threshold of 0.5 mm FD or 1.5 standardized spatial standard deviation of successive frames (DVARS) were annotated as motion outliers. The BOLD time‐series were then resampled into standard space using Lanczos interpolation in antsApplyTransforms, generating a preprocessed BOLD run in MNI152NLin6Asym space. All the pertinent transformations (i.e., head‐motion transform matrices and co‐registrations to anatomical and output spaces) were composed into a single resampling step. Independent component analysis (ICA) using FSL MELODIC (Beckmann and Smith 2004) was performed on the preprocessed BOLD in MNI space time‐series after removal of non‐steady state volumes and spatial smoothing with an isotropic, Gaussian kernel of 6 mm FWHM. Noise components were identified using ICA‐AROMA (Pruim et al. 2015).

Preprocessed fMRI data were reviewed for quality, and ICA‐derived noise components were manually identified by three expert raters (N.S. & M.A.M & G.B.P). ICs identified as noise by both raters were removed using MELODIC to generate an ICA‐denoised BOLD timeseries. We removed initial non‐steady state volumes from the denoised BOLD timeseries, then scaled the timeseries to a mean of 100.

### 2.5 Image Post‐Processing and Analysis

To generate activation maps associated with DBS cycling for each subject, we used 3dDeconvolve in AFNI to perform ordinary least squares multiple regression on the preprocessed and denoised fMRI data in MNI space. Each model included a boxcar regressor convolved with a standard hemodynamic response function corresponding to On and Off periods, as well as nuisance regressors for 3 polynomial drift terms. High motion timepoints (i.e., FD > 0.5 or DVARS > 1.5) were censored from analysis. Resulting parameter estimates corresponding to On and Off regressors were used to generate [On] – [Off] contrast maps for each individual run.

Group comparisons were performed using a 3dLME, a linear mixed‐effects (LME) model in AFNI, to compare On–Off changes in BOLD signal between therapeutic and nontherapeutic stimulation configurations. In the LME model, the responder status and therapeutic configurations were modeled as fixed effects and subjects were modeled as random effects. Cluster correction was applied on the group map using the following parameters: *p* = 0.05, alpha = 0.05, NN = 1 to yield a cluster size threshold of 2739.

### 2.6 DWI Processing and Electrode Reconstruction

Preprocessing was performed using standard methods in QSIPrep 0.15.2, which is based on Nipype 1.7.0 (Gorgolewski et al. 2011). DWI preprocessing in QSIPrep included the following steps: using MRtrix3, MP‐PCA denoising was applied to DWI volumes with dwidenoise (Veraart et al. 2016), Gibbs unringing was performed using mrdegibbs (Horn et al. 2019), and B1 field inhomogeneity was corrected with dwibiascorrect using the N4 algorithm (Tustison et al. 2010). Eddy current and head motion correction were performed using eddy (FSL 6.0.5.1) (Baniasadi et al. 2020), and eddy's outlier replacement was run with default parameters. Finally, DWI time‐series were resampled to the ACPC coordinate system and 1 mm isotropic voxels using the Jacobian modulation interpolation method in eddy (Veraart et al. 2016; Tustison et al. 2010; Kellner et al. 2016; Andersson and Sotiropoulos 2016).

We used Lead‐DBS 2.6 (Horn et al. 2019), a MATLAB toolbox for DBS electrode reconstruction and simulation of DBS, to model diffusion tracts stimulated by DBS. First, individual pre‐ and post‐operative T1w, T2w (where available), and DWI scans were co‐registered using SPM12 (Avants et al. 2008) and normalized to MNI152NLin2009b space using ANTs. Post‐operative T1w anatomical scans were used to manually localize DBS electrode locations. DBS electrodes were then reconstructed for each subject. Using FastField (Buckner and DiNicola 2019), which utilizes a volume conductor model of the DBS electrode and surrounding tissue, the volume of activated tissue (VAT) by stimulation was modeled for each bipolar stimulation configuration that was used during fMRI runs. We generated individualized whole‐brain tractography from denoised diffusion imaging data using the DSI studio implementation of the generalized q‐sampling (Yeh, Wedeen, and Tseng 2010) imaging method (GQI) in Lead Connectome. 200,000 fibers were estimated for each subject, using default parameters for the GQI tracking (Baniasadi et al. 2020). For each patient and bipolar stimulation configuration, whole‐brain tractograms were filtered to isolate streamlines that passed through the VAT. These remaining streamlines were used to estimate connectivity to parcels from Schaefer cortical (Schaefer et al. 2018) and Tian subcortical atlases (Tian et al. 2020).

### 2.7 Statistics for Network Analysis

The Schaefer 100‐parcel, seven network fMRI atlas was used to estimate the effect of the DBS On–Off response across these canonical networks for each individual fMRI run. We utilized a one‐sided bootstrap spatial permutation test to determine if there was a significantly increased BOLD suppression within one of the seven networks for the therapeutic and non‐therapeutic runs. To do this, we calculated the observed average amount of suppression across parcels within each of the seven networks for the therapeutic and non‐therapeutic runs. We then generated a surrogate distribution (*n* = 10,000) derived by permuting the parcels assigned to each of the seven networks to separately calculate the average amount of suppression within each network for the therapeutic runs and non‐therapeutic runs. *p*‐values were then calculated by finding the proportion of surrogates with suppressions greater than the actual differences observed, yielding one‐tailed *p*‐values for each network. Likewise, a similar two‐sided bootstrap method was used to calculate whether there was a significant difference in BOLD activation/suppression within each of the seven networks between the therapeutic and non‐therapeutic configurations. A Bonferroni correction was used to correct for multiple comparisons across the seven networks.

A similar approach using the Schaefer 100 parcel‐7 network fMRI atlas was used to quantify the structural connectivity as measured by the fraction of total streamlines from the estimated VAT to cortical parcels within the canonical networks. A similar one‐sided spatial bootstrap test was used to determine if there was a significantly increased total number of streamlines to one of the seven networks for the therapeutic configurations across responders and non‐therapeutic configurations across all subjects. This method was also used to determine if there was a significant difference in fraction of total streamlines between therapeutic and non‐therapeutic configurations in the seven networks.

## 3 Results

**FIGURE 1:**
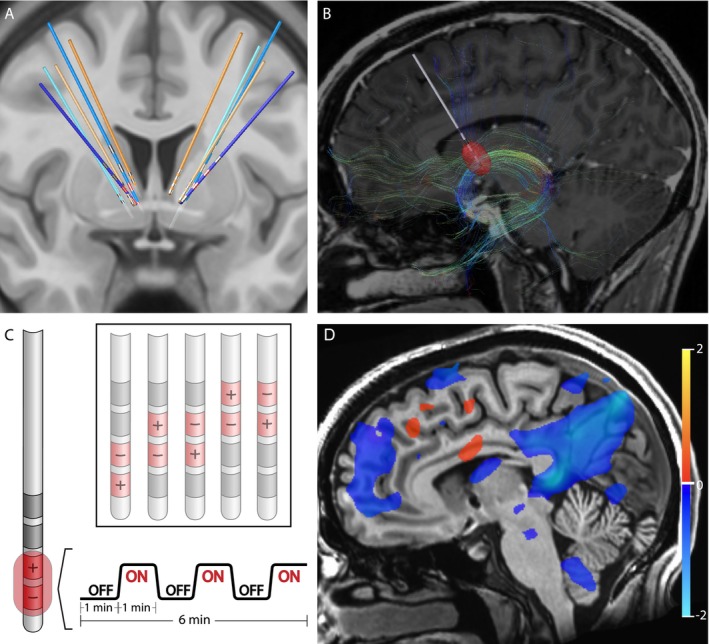
Structural and functional characterization of DBS configurations. (A) Reconstruction of DBS leads for five subjects. Leads for separate patients are in distinct colors. Blue leads indicate treatment responders; orange leads indicate non‐responders. Therapeutic electrode contacts shown in red. (B) Example of tractography derived from diffusion imaging seeding the estimated volume of tissue activation for a therapeutic bipolar contact configuration for a single representative subject. (C) Design of DBS cycling On versus Off paradigm during fMRI acquisition for different stimulation configurations. (D) fMRI BOLD changes with DBS On versus Off for same subject and configuration in (B). Suppression of BOLD with DBS On versus. Off is depicted in blue while activation with DBS On versus Off is depicted in red. Color bar indicates percentage change in BOLD signal. *p* < 0.05; OLSQ.

**FIGURE 2:**
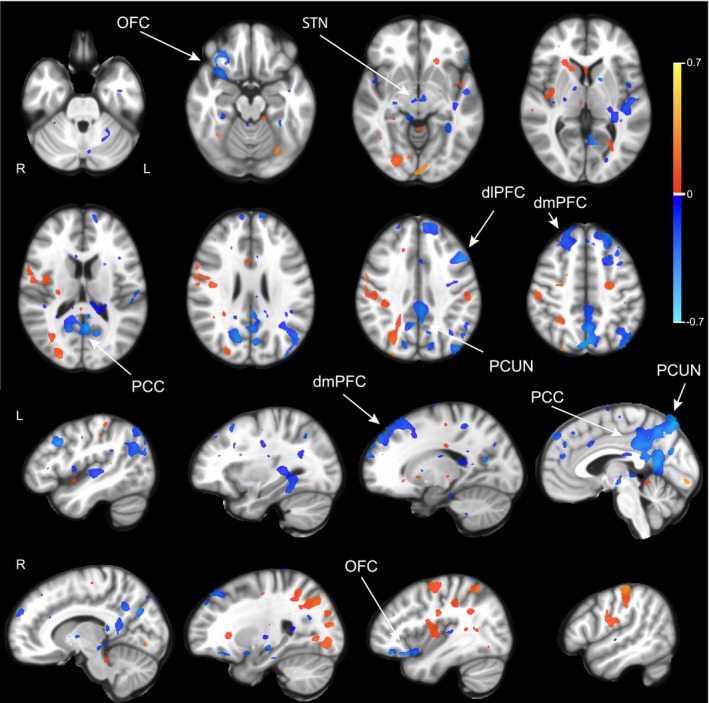
BOLD response differences between therapeutic and non‐therapeutic DBS. Group comparison using linear mixed effects model of BOLD response between DBS On and Off in therapeutic (*n* = 6 runs, 3 subjects) versus nontherapeutic (*n* = 17 runs, 5 subjects) DBS configurations. Activations are in red and suppressions in blue. Color bar indicates percentage change in BOLD signal. dmPFC, Dorsomedial prefrontal cortex; OFC, Orbitofrontal cortex; PCUN, Precuneus; PCC, Posterior cingulate cortex; STN, Subthalamic nucleus. *p* < 0.05; LME.

Five patients who had been treated with DBS for their severe, refractory OCD were identified from the UCSF OCD Clinic. Their DBS leads were located within the ALIC and neighboring BNST (Figure 1A, Figure S1). For each of the patients, structural and diffusion‐weighted MRI data were collected to identify the structural connectivity from the estimated volume of activated tissues (VAT) from different electrode stimulation configurations (Figure 1B). fMRI data were acquired at 3 Tesla while the DBS device was set in a one‐minute ON/one‐minute OFF cycling paradigm (Figure 1C). Subsequently, DBS On‐vs‐Off contrast maps were generated for each DBS configuration (Figure 1D). Group contrast maps between DBS On versus Off for the therapeutic (Figure S2) and non‐therapeutic (Figure S3) configurations were derived. For therapeutic DBS configurations, we identified significant suppression in components of the CSTC circuit implicated in OCD such as the right orbitofrontal cortex, bilateral dorsomedial prefrontal cortex, left subthalamic nuclei, and right thalamus as well as other regions, such as the precuneus and posterior cingulate cortex (Figure S1, *p* < 0.05 LME). For non‐therapeutic DBS configurations, we found heterogenous changes, which were often not consistent across configurations or participants (Figure S2, *p* < 0.05 LME). We also generated a difference map between the therapeutic versus non‐therapeutic configurations for DBS On‐vs‐Off, which identified significant BOLD suppression in the right orbitofrontal cortex, bilateral dorsomedial prefrontal cortex, and bilateral subthalamic nuclei, components of the CSTC network, as well as the left dorsolateral prefrontal cortex, precuneus, and posterior cingulate cortex (Figure 2, *p* < 0.05, LME; Figure S4). In general, suppressions of BOLD activity for therapeutic configurations with DBS On‐vs‐Off, and the difference map between therapeutic and non‐therapeutic configurations, corresponded to regions distant from the sites of the active electrode contacts located in the ALIC. However, it is possible that local BOLD signal changes within the ALIC itself may have been obscured by the presence of the electrode artifact.

**FIGURE 3:**
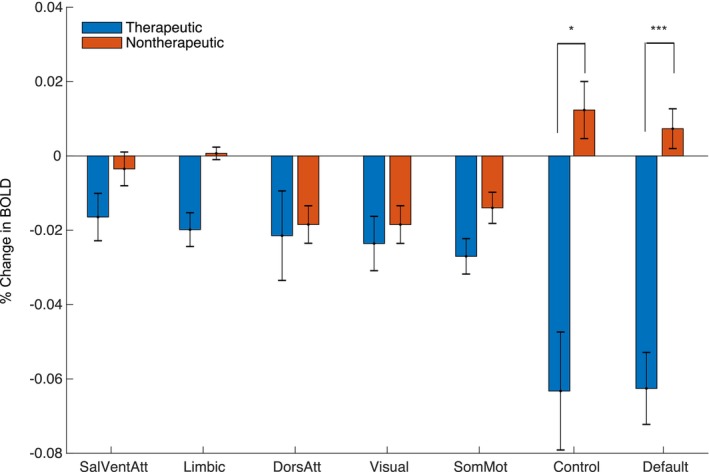
Network Impact of Therapeutic and Non‐Therapeutic DBS. Comparison of average BOLD changes within canonical resting‐state networks for therapeutic (blue) and non‐therapeutic (red) configurations. ***p < 5.0 × 10^−3^, *p < 0.05; permutation test (one‐sided with Bonferroni correction).

Next, we asked whether the observed pattern of DBS On‐vs‐Off BOLD responses localized to particular functional networks by extracting network‐specific parameter estimates for each contrast using existing network atlases (Figure S5). Comparing DBS On‐vs‐Off for therapeutic configurations revealed a significant decrease in BOLD signal within the default mode network (DMN) (*p* = 1.0 × 10^−4^, one‐sided permutation test with Bonferroni correction). We also observed a significant difference in BOLD signal change between therapeutic and nontherapeutic DBS On‐vs‐Off in the DMN (*p* = 1.0 × 10^−4^, one‐sided permutation test with Bonferroni correction) and control network (*p* = 2.2 × 10^−3^, one‐sided permutation test with Bonferroni correction) (Figure 3).

**FIGURE 4:**
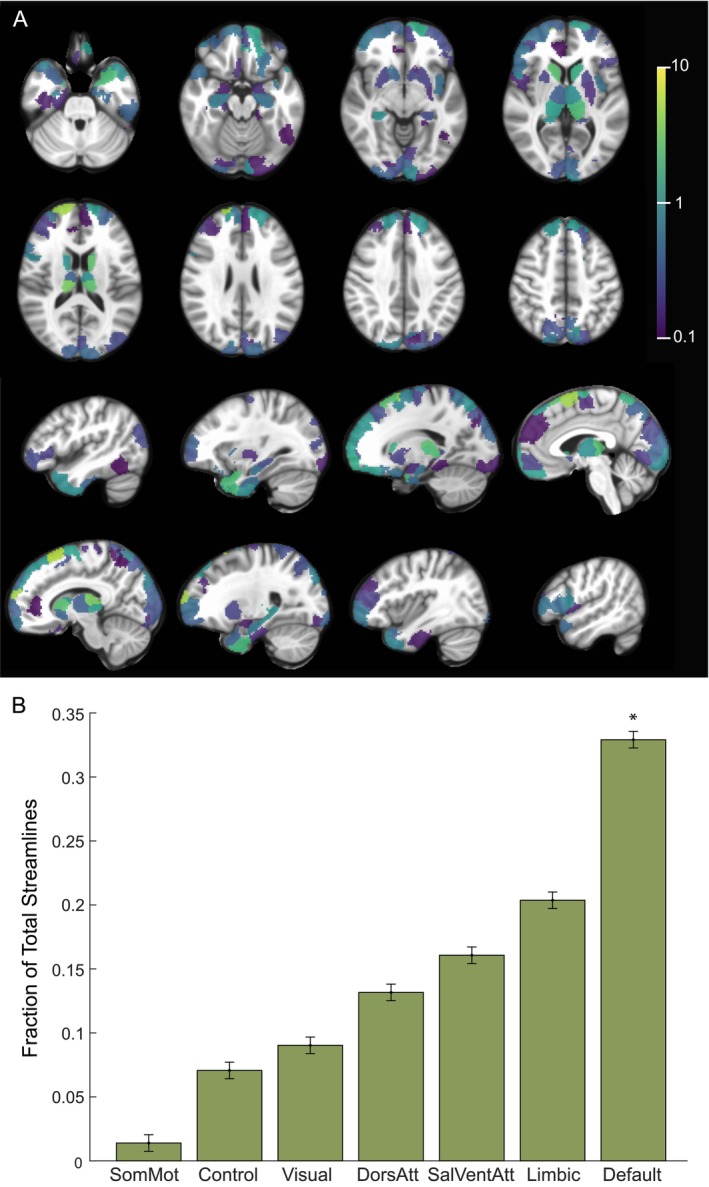
Structural Connectivity with Therapeutic Electrode Configurations. (A) Percentage of total streamlines from the estimated volume of activated tissue to functional network parcels for the therapeutic configurations (*n* = 6 runs, 3 subjects). (B) Resting state networks ordered based on increasing fraction of streamline counts from the estimated volume of activated tissue for the therapeutic configurations. *p < 0.05, permutation test (one‐sided).

Finally, we sought to determine whether structural connectivity from the estimated VAT for each DBS configuration identified similar functional networks by comparing the fraction of streamlines reaching each network parcel (Figure S6). For the therapeutic configurations, we found significantly increased fraction of structural connections to the DMN relative to other networks (Figure 4A,B; *p* = 0.011, one‐sided permutation test). We also found that therapeutic configurations had significantly more structural connectivity to the limbic network compared to non‐therapeutic configurations (*p* = 4.2 × 10^−3^, two‐sided permutation test with Bonferroni correction).

## 4 Discussion

In this study, we developed an fMRI paradigm in which DBS is cycled On and Off to investigate the brain network mechanisms underlying this treatment. We found that DBS from therapeutic contacts induced long‐range BOLD suppression in regions implicated in OCD such as the lateral orbitofrontal cortex, dorsomedial prefrontal cortex, and subthalamic nuclei. Many of these suppressions were found to be concentrated within the DMN. In contrast, DBS configurations that were non‐therapeutic often led to heterogenous and non‐specific brain activation patterns. Moreover, we found that the estimated VAT nearby therapeutic DBS contacts showed significant structural connected to the DMN, but not to other networks.

Based upon these findings, we propose a model in which therapeutic ALIC DBS operates by interrupting communication through white matter to structurally connected regions such as the medial prefrontal cortex, lateral orbitofrontal cortex, thalamus, and midbrain, which are part of a CSTC circuit implicated in OCD. The suppression of these directly connected regions in turn leads to a much wider cascading set of suppressions throughout the wider DMN including regions that do not appear have direct structural connections to the leads, presumably by way of polysynaptic connections. The DMN is often associated in the literature with internalizing states (Buckner and DiNicola 2019), and prior studies have implicated disruptions in functional connectivity within the DMN (Goncalves et al. 2017) and between the DMN and other networks in the pathophysiology of OCD (Posner et al. 2017). We speculate that the suppression of DMN activity might mediate the therapeutic effects of ALIC DBS by reducing obsessions and other internalizing states. Likewise, prior studies have noted that excessive activity within the frontoparietal control network may be associated impairments in cognitive flexibility in OCD (Liu et al. 2023; Gruner and Pittenger 2017; de Vries et al. 2014). It is possible that suppression of the neighboring control network with ALIC DBS also may be associated with improvements in compulsive behaviors.

Many components of the CSTC circuit suppressed by DBS On‐vs‐Off are neuromodulation targets for treating OCD. For example, we observe DBS suppression of dorsomedial PFC (Carmi et al. 2019) and orbitofrontal cortex (Williams et al. 2021; Nauczyciel and Drapier 2012), which are transcranial magnetic stimulation targets in OCD. Similarly, the suppression observed in subcortical structures such as the subthalamic nucleus (Mallet et al. 2019) are of interest given that this region is also an alternative DBS target for treating OCD. This pattern of functional suppression also mirrors structural connections whose stimulation has been associated with improved DBS outcomes for OCD (Baldermann et al. 2021; Li et al. 2021; Li et al. 2020; Mosley et al. 2021). By contrast, we observe that non‐therapeutic configurations fail to exert the same disruptive effect on the DMN and its associated subcortically connected circuits. In contrast, many of the non‐therapeutic stimulation configurations instead appear to enhance, rather than suppress, brain‐wide activity. Prior positron emission tomography (PET) studies have observed heterogenous effects including activations (Rauch et al. 2006) as well as suppressions (Van Laere et al. 2006) in components of the OCD CSTC network with stimulation. However, these studies did not differentiate between therapeutic and non‐therapeutic configurations in their study design.

The fact that similar DBS parameters can have opposing functional effects within and across distributed circuits is striking. These findings are not easily reconciled with existing models which posit that DBS stimulation always behaves like a functional lesion within a restricted anatomical region or structurally connected circuit. Instead, they suggest that the impact of DBS on distributed circuits may be activating or suppressing depending on a wide variety of factors, including individual differences in anatomy, network state, and other patient‐specific factors. For example, recent studies have suggested that there are tracts (Li et al. 2020) and anatomical regions (Meyer et al. 2024) within the ALIC region that may be associated with better or worse outcomes, and these targets may be associated with differential effects on activating or suppressing network activity. Indeed, a study in a rodent model of DBS for OCD described similar competing neural populations within the same region in response to ALIC DBS, pointing to the complex effects that neurostimulation can have on downstream circuits (van den Boom et al. 2023). Our findings are also consistent with results in Parkinson's disease, demonstrating that therapeutic STN DBS appeared to decrease BOLD activity within a motor network whereas non‐therapeutic DBS seemed to recruit non‐specific activity in other non‐motor regions (Boutet et al. 2021). Prior studies have also suggested that the clinical effect of stimulation may be dependent on factors such as the current symptom state (Scangos et al. 2021a), motivating the need to develop closed‐loop strategies for neuromodulation (Scangos et al. 2021b; Oehrn et al. 2024).

There are several limitations to this study. First, the sample includes only a small number of patients. However, by leveraging the fact that multiple DBS configurations could be trialed and compared within each subject, we were nevertheless able to identify changes in network activity that were specific to therapeutic DBS. We also utilized a block design, which can yield a larger effect size than alternative study designs. Another limitation is that we could not consistently observe the local impact of DBS using fMRI due to the presence of the electrode artifact around the stimulation contacts. Lastly, it is unclear how the acute functional changes that we observe using our DBS On/Off cycling protocol are related to the long‐term OCD benefit of continuous stimulation. It is possible that the suppression of the DMN with cycling DBS On and Off may be more related to acute mood and anxiety changes, rather than changes specific to the core pathophysiology of OCD (Gibson et al. 2017). Indeed, during testing for tolerability, the DBS programming clinician noted that the therapeutic DBS in our responders was uniformly associated with acute improvements in mood and reductions in anxiety. In contrast, many of the non‐therapeutic settings were associated with worse anxiety following stimulation.

Our study suggests that therapeutic DBS suppresses the CSTC circuit and DMN in responders. Future studies will be needed to deterposte if the same acute network changes that we observe with therapeutic ALIC DBS can also be observed with other evidence‐based DBS targets for OCD, such as the anteromedial subthalamic nuclei and how they relate to proposed anatomic sweet‐spots for OCD DBS within the ALIC region (Meyer et al. 2024). It also remains to be seen whether the use of imaging‐based biomarkers can help guide and simplify the process of DBS programming. Our results may also inform other closed‐loop approaches targeted to suppress CSTC circuit activity to improve outcomes for patients with severe, refractory OCD (Provenza et al. 2021).

## Author Contributions

Conception and design of the work: A. Moses Lee and Melanie A. Morrison. Acquisition of data: A. Moses Lee, Natalya Slepneva, Melanie A. Morrison, Adam C. Frank. Analysis and interpretation of the data: Natalya Slepneva, Melanie A. Morrison, A. Moses Lee, Genevieve Basich‐Pease. Writing of manuscript: A. Moses Lee, Natalya Slepneva, Melanie A. Morrison. Resources: A. Moses Lee and Melanie A. Morrison. Supervision: A. Moses Lee and Melanie A. Morrison.

## Conflicts of Interest

The authors declare no conflicts of interest.

## Supporting information


Data S1.

## Data Availability

The data that support the findings of this study are available on request from the corresponding author. The data are not publicly available due to privacy or ethical restrictions.
